# Pain Management in Burned Patients Treated with Bromelain-Based Enzymatic Debridement

**DOI:** 10.3390/jcm14051571

**Published:** 2025-02-26

**Authors:** Michelle Laurens Acevedo, Gemma M. Usua, Juan P. Barret

**Affiliations:** 1 Department of Anaesthesiology, Vall d’Hebron Barcelona Hospital Campus, 08035 Barcelona, Spain; 2 Department of Plastic Surgery and Burns, Vall d’Hebron Barcelona Hospital Campus, 08035 Barcelona, Spain; 3 Department of Surgery, School of Medicine, Universitat Autònoma de Barcelona, 08035 Barcelona, Spain

**Keywords:** burn, pain, bromelain, enzymatic debridement

## Abstract

**Background/Objectives:** Enzymatic debridement with bromelain is a treatment option for deep partial thickness and full thickness burns. This procedure is associated with significant pain, necessitating the use of anesthesia techniques. However, there is limited evidence on the optimal strategy to achieve effective pain control. To detail the anesthetic approach in patients undergoing bromelain-based enzymatic debridement for burn injuries. **Methods:** A retrospective observational study was conducted by analysing the medical records of burn patients treated with enzymatic debridement using bromelain. The study included patients admitted to the Burn Unit of Vall d’Hebron University Hospital between January 2015 and December 2019. **Results:** A total of 112 patients met the inclusion criteria. The average burned total body surface area (TBSA) was 10.7% ± 11.4, and the median Abbreviated Burn Severity Index (ABSI) was 5 (range: 2–12). The most commonly burned and treated regions were the upper limbs (73%), followed by the lower limbs (30%) and the abdomen (8%). Regional anesthesia was the predominant technique, utilised in 96% of cases. Among these, axillary nerve block was performed in 47% of patients, with continuous catheter placement in 31%. Pain control was achieved in 61% of patients during the first 48 h following enzymatic debridement. Opioids were required for post-procedure pain relief in 12.5% of cases, and repeat anesthesia was necessary in 2.7%. There was no significant difference in pain management outcomes between single nerve blocks and catheter-based approaches (*p* = 0.809). Complications were reported in nine patients and included hypotension, nausea, and urinary retention. **Conclusions:** Bromelain-based enzymatic debridement is a painful intervention requiring specialised anesthetic management. Regional anesthesia techniques offer a safe and effective strategy for pain control, though achieving optimal analgesia during the initial 48 h remains a clinical challenge.

## 1. Introduction

Eschar removal within the first 72 h in burned patients has shown to improve functional and aesthetic outcomes, reducing infection and hospital stay [1]. Surgical debridement is the standard procedure, but it can be traumatic and is not exempt from complications, entails inadvertent resection of viable tissue, and facilitates blood and heat loss. In addition, it requires specific surgical equipment and qualified personnel, so there is a necessity to find other alternatives for burned patients [2,3].

Bromelain-based enzymatic debridement (Nexobrid^®^, MediWound, Yavne, Israel) is a recent technique that allows prompt debridement for burn patients. It is a plant-based medication that was approved by the European Medicines Agency in 2012 [4,5]. It consists of a mixture of proteolytic proteins from pineapple stems that contains the enzyme bromelain. It has been approved in adults for eschar removal of deep partial and full thickness thermal burn injuries [4,5]. It has documented advantages compared with surgical debridement, including the reduction of debridement time; leaving clean surgical zones that have enough epidermis for re-epithelisation; and reducing the need for surgery and autografts, infection, blood loss, and length of hospital stay [1,2,6].

Even though enzymatic debridement is a good option for eschar removal [3,7], it has some issues, such as coagulation disorders [8]. Furthermore, it is a painful procedure that should be performed under pain management protocols [1,9]. Some of the recommendations suggest using regional anaesthesia, with or without the placement of perineural catheters with a long-lasting anaesthetic or general anaesthesia when burns are both on the trunk and extremities [1,10,11]. Nevertheless, most of the recommendations are based on expert consensus and the clinical experience of case reports. This study provides a comprehensive analysis of the anaesthetic techniques utilised during the ED with Nexobrid^®^ in our unit. We present our experience with the pain management in ED treatment in burned patients, describing the anaesthetic strategies in burn patients that underwent debridement with Nexobrid^®^ to help standardise proper pain management strategies in ED with Nexobrid^®^ in burned patients.

## 2. Patients and Methods

We conducted a retrospective observational study in burn patients treated with Nexobrid^®^ that were admitted to Vall d’Hebron University Hospital from January 2015 to December 2019. We included patients older than 18 years that were assessed by the anaesthesia team and had deep partial or full thickness thermal burns. Exclusion criteria were patients with electrical or chemical burns and superficial or superficial partial thickness burns, as well as patients in which the Anaesthesia Department did not participate actively in their pain management that were on sedation and mechanical ventilation due to their active illness process.

For the data collection, we performed a review on the electronic clinical chart of the included patients. In our centre, all patient-related data are stored in the electronic hospital database of patients. For special units, such as our Burn Centre, special data storage for the Burn Patient Clinical Process (Procés Pacient Cremat) exists and is available for review. Demographic variables included age, sex, comorbidities, percentage of the total body surface area (%TBSA) burned, burn location, and aetiology. In addition, we collected information related to the anaesthesia procedures performed for pain control, complications derived from the intervention, the need to repeat an anaesthetic technique, and the use of opioids as a rescue treatment alternative.

### 2.1. Procedure Description

At our institution, Nexobrid^®^ is applied after a pre-operative assessment, with informed consent and following fasting guidelines [12]. Following our centre’s protocol and the manufacturer’s guidelines, ED is performed within the first five days post-injury. First, the anaesthesia team performs the technique that is considered appropriate to the case and the burned region. A nerve block involves the injection of an anaesthetic next to a nerve or group of nerves, which numbs the entire region innervated by those nerves. It is usually performed to anaesthetise the hands, feet, arms, legs, groin, or specific areas of the face. The patient is conscious during the procedure to be performed but has no pain in the area. Regional anaesthesia produces less nausea and allows for a quicker recovery than general anaesthesia. It also provides more effective pain control after surgery than the administration of narcotic medications through a vein. Different nerves blocks are generally used depending on the area where the burn wound is to be treated, such as a brachial plexus block. This is required for operations on the shoulder or arm. Depending on the specific surgical site, one body region or another will be numbed. If surgery is to be performed on the shoulder or collarbone, the nerves may be blocked above the collarbone; if surgery is to be performed on the arm, they may be blocked in the armpit area.

Paravertebral blocks are performed on the sides of the spine, where the nerves exit from the spinal cord. The nerves are blocked at the neck level for thyroid or carotid artery surgery, at the back level for various operations on the chest or abdomen, and at the hip level for operations on the hips, thighs, or knees. A femoral nerve block is commonly used for knee operations by injecting at the groin level. The sciatic block is used for knee, calf, Achilles tendon, ankle, or foot surgery. In some patients, nerve blocks may be combined with i.v. sedation to aid in the post-procedural pain.

Once it is performed, the nursing staff pre-soaks the burn injury, which helps to identify the effectiveness of the anaesthesia technique. Blisters are removed, and devitalised skin is mechanically cleared. The wound is then covered with an occlusive moist dressing containing Prontosan^®^ (B. Braun, Germany) for at least two hours to prepare for debridement. To start the procedure, the dressing is removed, and the burn area is outlined with sterile petrolatum After this, Nexobrid^®^ is applied and covered for 4 h. Then, it is removed by the drag technique and washed energetically. Finally, the wound is covered again until the next day when it is examined by the plastic surgery team. The whole procedure can be painful, which is why the anaesthesia strategy should be long-lasting to offer better comfort to the patient. Pain management includes sedoanalgesia or regional nerve blocks.

Nexobrid^®^ was applied in a layer of 1.5–3 mm, covered by an occlusive dressing. Four hours later, the dressing was removed, and the area was cleansed with physiological saline using a spatula. A moist dressing with Prontosan^®^ (B. Braun Medical S.A.U, Rubí, Spain) was reapplied for at least six hours until the next morning’s post-debridement evaluation.

In our unit, Nexobrid^®^ is used for deep partial and full thickness thermal burns located on the head and neck, extremities, trunk, and abdomen. It is important to mention that we do not apply it to patients with more than 15% TBSA burned according to medication regulations and current guidelines [1,13]. In addition, not all burn wounds in one patient can be treated by this medication, so we prioritise those regions that are highly functional and can benefit from enzymatic debridement.

### 2.2. Outcome Measurement

The principal outcome in our study was pain control during the first 48 h after the application of Nexobrid^®^. Data were obtained from the clinical charts registered by the nursing staff.

### 2.3. Statistical Analysis

We conducted a descriptive analysis, calculating frequencies and percentages for qualitative variables, as well as means and medians for quantitative variables. Measures of dispersion, including standard deviation and interquartile range, were applied as appropriate. Post-operative pain and %TBSA burned were compared. %TBSA burned was subdivided into two groups, one with patients presenting with a %TBSA burned of 10% or lower and another with %TBSA% burned greater than 10%. Post-operative pain and number of affected areas were also compared, dividing patients with a single affected area from those with multiple affected areas. Post-operative pain and burn aetiology, sex, age, and single nerve block vs. catheter placement were also assessed. Pearson’s chi-squared test was employed for inferential analysis, as the majority of our data consisted of qualitative variables. A *p*-value of less than 0.05 (*p* < 0.05) was deemed statistically significant.

### 2.4. Ethical Considerations

This research was approved by the Ethics Committee of the Vall d’Hebron University Hospital (reference number: PR(AG)161/2020). The certification was issued on 6 May 2020. In addition, it was approved by the Spanish Agency of Medicines and Medical Devices (AEMPS) (LML-BRO-2020-01).

## 3. Results

**Figure 1:**
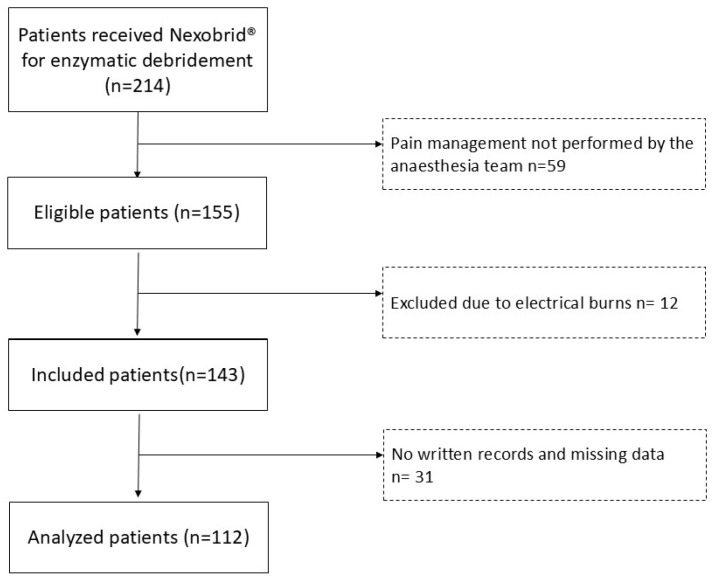
Study flow chart.

During the time of the study, 214 patients received Nexobrid^®^ for enzymatic debridement in our burn unit. Of these, 59 patients were excluded because pain management was not performed by the anaesthesia team. They were patients admitted to the Burn Intensive Care Unit (ICU) that required mechanical ventilation and sedation, and their pain management was addressed by Burn ICU intensivists. Out of the remaining 155 patients, 12 patients were not enrolled because they had electrical burns and 31 patients were excluded because there were no written records during the use of Nexobrid^®^. Thus, we had 112 eligible patients for the study (Figure 1).

In this population, 75% were male, with a median age of 45.3 years (SD 17.8) and 43.4 years (SD 17.8), respectively. In addition, 13.9% of the patients had two or more comorbidities as described in Table 1. The mean %TBSA burned was 10.7% (SD 11.3, CI 0–32.9%), and the median for the Abbreviated Burn Severity Index (ABSI) was 5 (IR 2–12). In more than two-thirds of the patients, flame was the mechanism of the burn injury (71%, SD 4%, CI 63–79%), followed by scald (30%, SD 4%, CI 22–38%) and contact (3%, SD 1%, CI 0–5%).

The upper and lower limbs were the most frequent parts of the body that were compromised in our study population (83% and 60%, respectively). Notably, not all burn injuries in a single patient were treated. In our protocol, burned regions located in functional areas were prioritised over others to receive enzymatic debridement. In addition, we had zero reports of patients with head and neck injuries and back and trunk injuries treated with Nexobrid^®^ (Table 2). This is because they were admitted to the ICU on mechanical ventilation, and their pain management was performed by ICU teams, meeting exclusion criteria.

Regional anaesthesia was the most common approach to pain control in our study (96% of the cases; SD 2%, CI 92–99%). Peridural catheter insertion was the most common neuraxial technique performed (27%, SD 4%, CI 19–35%), and axillary nerve block was the most frequent peripheral technique performed (47%, SD 5%, CI 38–56%) (Table 3). Catheter placement during the technique was performed in 31% of the cases (SD 4%, CI 22–39%). Only nine patients had complications derived from anaesthesia management, including eight patients with hypotension, three patients with nausea and vomiting, and one patient with urinary retention.

### Pain Control

In our study, 61% of patients experienced no pain within the first 48 h following enzymatic debridement. Opioids were administered as a rescue treatment for pain relief in 12.5% of cases, while the anesthesia technique had to be repeated in 2.7% of cases due to insufficient effectiveness. There were no differences in pain control in the first 48 h when a comparison was made between patients receiving a single nerve block and those receiving catheter placement (*p* = 0.809). Opioid use in patients with continuous infusion of local anaesthetics comprised 38.5% of the cases compared to 61.5% of the cases with single nerve block; however, the difference was not statistically significant (*p* = 0.319). Notably, there were no differences in pain control between the patients that had all their injuries treated with Nexobrid^®^ compared to those who did not have all their injuries treated with Nexobrid^®^ (*p* = 0.869). We found a correlation between age and pain. In the group of patients under 30 years old, 53.7% of them reported pain after the procedure compared to 31.2% of the patients that were over this age (*p* = 0.035). There was no relation between a higher incidence of pain and sex or aetiology of the burn (Table 4).

## 4. Discussion

Enzymatic debridement is described as a very painful procedure, in which invasiveness should not be underestimated [1,2]. There are no validated protocols to provide analgesia during and after the procedure [9]. Evidence is based on expert consensus [1,10] and the clinical experience of case reports [11]. Our study included 122 patients, which makes it the largest sample size of patients reported to date.

According to previous studies, regional anaesthesia seems to be superior for pain management compared to sedation [3,11,14]. In our experience and following the recommendations of the clinical guidelines, most of the patients received regional anaesthesia techniques [1,9]. We documented appropriate pain control in the first 48 h in almost two-thirds of the population. However, 39% of the patients reported pain during the first 48 h after debridement. Notably, only 12.5% of the patients needed opioids for pain relief, suggesting that these patients had moderate to severe pain. One possible reason for this is that some patients had multiple burned regions and not all of them received debridement with Nexobrid^®^. Therefore, they were not covered by regional anaesthesia techniques. Because there was not enough information in the clinical charts, we could not differentiate if the pain reported was over the area debrided or the other burned regions. In addition, pain in burn patients can be underestimated and techniques such as patient-controlled analgesia are recommended [15], since rescue analgesia by nurses on the surgical ward may be less systematic [16]. In third place, anxiety and pain in burn patients are strongly associated and are perceived in a similar manner [17,18]. We did not use standardised strategies to approach the management of anxiety in our patients, which could influence our pain relief results.

When comparing between a single peripheral nerve block and catheter placement, there were no differences in pain control between these techniques. This is surprising, since based on reported experience, patients had severe pain as the single peripheral nerve block wore off, requiring long-acting rescue opioids [14]. Capdevila et al. reported a multicentre prospective analysis with continuous peripheral nerve block after orthopaedic surgery, finding that patients had increased pain after 24 h of the anaesthesia technique [16]. These results are comparable to our data. One possible explanation is that low-volume infusions or low concentrations of local anaesthetics may be insufficient to achieve adequate pain relief when compared to the high volume and high concentrations used in the initial bolus after catheter placement or in the single nerve block technique [16,18]. In addition, we were not able to differentiate if the pain reported by our patients was over the area treated with Nexobrid^®^ or a different injured region, which could influence the results when comparing a continuous or single peripheral nerve block. More prospective studies are needed to evaluate whether one strategy is better than the other.

Notably, Galeiras et al. reported a protocol based on procedural sedation and analgesia in 17 patients treated with Nexobrid^®^ [9]. They documented, through the Numeric Pain Rating Scale (NPRS), pain above 4 in 17.6% of the cases. These results were reported only during the procedure. Buta et al. reported an NPRS score of 3.6 24 h after the procedure [19]. There is no evidence regarding pain management following enzymatic debridement, so it is unknown whether it is correctly approached and treated in the following days. Our data suggest that it is underestimated, and there is a necessity to find better strategies to offer pain relief in the first 48 h after the application of Nexobrid^®^.

Most of the patients included in our research had burn injuries of the extremities. This is because, in our institution, Nexobrid^®^ is a medication that is preserved for specific cases where functional areas of the body are affected. Based on this, most of the patients were suitable for regional anaesthesia techniques [20,21]. In cases where there was more than one area burned, the team prioritised functional areas in the extremities. Notably, there were no differences in pain control between the patients that had all their injuries treated with Nexobrid^®^ when compared to those who did not have all their injuries treated with Nexobrid^®^. These findings might be related to the low number of patients in the study and the missing data from the clinical charts. In addition, these results highlight the complexity in the management of pain in burn patients, making it necessary to carry out more research studies for the correct approach [22,23].

There are some limitations in our study. First, we could not quantify by objective scales the intensity of the pain in the patients during the first 48 h after the enzymatic debridement. The results are based on the presence or absence of pain as reported in the clinical history, because the information was gathered by clinical chart review where these data were missing. Second, we could not identify if the pain reported in the charts was in the area treated with Nexobrid^®^ or in a different region in patients with multiple burn injuries. As mentioned before, this information was also recorded in the clinical charts, implying an important bias in our results. Finally, we did not approach the pain management in patients that underwent the procedure and were already on mechanical ventilation and sedation. These patients have a more complex pain control assessment, and there was no information as to whether it was approached correctly. 

## 5. Conclusions

Bromelain-based enzymatic debridement is a highly painful procedure that necessitates advanced and specialised anesthesia management. Based on our experience, regional anesthesia is the most commonly employed technique for pain control, aligning with recommendations from published data. However, there were many patients that had poor pain control in the first 48 h after enzymatic debridement. We could not find clear differences in pain relief with continuous nerve blocks over single shots. More studies are needed to assess the best strategy for analgesia during and after the application of Nexobrid^®^.

## Figures and Tables

**Table 1:** Demographic characteristics of the studied population.

Characteristic	N	%
Sex Male Female	80 27	75% 25%
Age, years, mean (SD) Male Female	45.35 (17.75) 43.37 (17.78)	
Comorbidities Hypertension Diabetes Mellitus Mental illness Drug abuse Heart disease Chronic Kidney Disease Obesity Coagulopathy	19 (3) 9 (2) 6 (2) 16 (3) 4 (2) 3 (1) 3 (1) 4 (2)	16% 8% 5% 14% 3% 3% 3% 3%
Etiology Flame Scald Contact	83 (4) 35 (4) 3(1)	71% 30% 3%
Burn %TBSA, mean (SD)	10.7% (11.34%)	

**Table 2:** Description of the burn injury location. In the second row, the number of patients that had a specific burn location is shown. In the third row, the burned locations that were treated with Nexobrid ^®^ are shown.

	Burned	Burned and Treated
Head and neck	47 (40%)	0 (0%)
Upper limb	98 (83%)	71 (73%)
Front trunk	23 (19%)	1 (5%)
Back trunk	5 (4%)	0 (0%)
Abdomen	12 (10%)	1 (8%)
Lower limb	71 (60%)	50 (70%)

**Table 3:** Description of the anaesthesia techniques performed in the study.

Anaesthesia Technique	N	%
Sedation	64	54%
Regional Anaesthesia	113	96%
Neuraxial Anaesthesia Spinal anaesthesia Epidural Anaesthesia	2 32	2% 27%
Peripheral nerve block Interscalene nerve block Supraclavicular nerve block Infraclavicular nerve block Axillary nerve block Femoral nerve block Sciatic nerve block	5 7 3 56 10 13	4% 6% 3% 47% 8% 11%
Catheter Placement	36	31%

**Table 4:** Comparison of variables with pain in the first 48 h after enzymatic debridement.

Characteristic	Pain	*p* Value
Sex Male Female	39.3% 37.9%	0.894
Age, years, mean (SD) Less than 33 years old 33 to 53 years old More than 53 years old	53.7% 36.8% 25.6%	0.035
Regional Anaesthesia Single nerve block Catheter placement	39.0% 38.9%	0.809
Etiology Flame Scald Contact	44.3% 28.1% 33.3%	0.518
Injuries treated with Nexobrid® All burned injuries treated Not all burned injuries treated	37.5% 40.0%	0.784

## Data Availability

The raw data supporting the conclusions of this article will be made available by the corresponding author, JPB, upon reasonable request.
